# Trifunctional antibody-cytokine fusion protein formats for tumor-targeted combination of IL-15 with IL-7 or IL-21

**DOI:** 10.3389/fimmu.2025.1498697

**Published:** 2025-04-30

**Authors:** Annika M. Möller, Sarah Vettermann, Felix Baumann, Max Pütter, Dafne Müller

**Affiliations:** Institute of Cell Biology and Immunology, University of Stuttgart, Stuttgart, Germany

**Keywords:** trifunctional, antibody-cytokine fusion proteins, common gamma chain receptor family, IL-15, IL-7, IL-21, cancer immunotherapy

## Abstract

Cytokines from the common gamma chain receptor family, such as IL-15, IL-21 and IL-7, show promise for cancer immunotherapy and have been incorporated individually into the immunocytokine approach. However, their efficacy as monotherapy is limited. Here, we investigated the molecular design of tumor-directed trifunctional antibody-cytokine fusion proteins for a combinatorial approach of IL-15 with either IL-7 or IL-21. Various fusion proteins differing in antibody format, cytokine composition and arrangement were generated and cooperative cytokine activity assessed in solution and bound to target cells. Comparative analysis revealed that formats with cytokines positioned at the N- and C-termini of the antibody were more effective than those arranged in series. For the former design, cooperative effects were observed with the scFv-based (IL-15+IL-7) trifunctional fusion protein, primarily enhancing the proliferation of naive T cells, while the scFv/Fab-based (IL-15+IL-21) trifunctional fusion proteins enhanced IFN-y release and the cytotoxic potential of T cells. Combining cytokines in the two-in-one molecule approach was principally advantageous when bound to target cells. Greater potency in inducing JAK-STAT pathway activation highlighted the importance of cytokine colocalization for cooperative receptor activation. Compared to the Fab-based (IL-15+IL-21) format, the scFv-based (IL-15+IL-21) format displayed a tendency towards higher activity in targeted and lower activity in untargeted settings, emphasizing the targeted concept. Thus, this study underscores the importance of molecular design in developing trifunctional immunocytokines and identified the scFv-based trifunctional (IL-15+IL-21) fusion protein, with the antibody in the central position, as a particularly promising candidate for further drug development.

## 1 Introduction

Cytokines of the common gamma chain receptor family play an important role in the response and homeostasis of lymphocytes, holding great potential for cancer immunotherapy (1). Next to IL-2, the most investigated member of the family, IL-15 has emerged as particular promising candidate in expanding and enhancing the function of lymphocytes important for an antitumor response (2). IL-15 induces the proliferation and differentiation of CD8+ T cells, supports the survival of CD8+ memory T cells, and is also involved in the generation, proliferation, and activation of natural killer (NK) cells (3). Unlike IL-2, IL-15 rather inhibits activation-induced cell death (AICD) and renders resistance to regulatory T cell function (4, 5). Under physiological conditions, IL-15 is usually presented in trans by its IL-15Rα chain to neighboring cells expressing the IL-15Rβγ chain constellation (6). Current IL-15 drug developments mostly integrate parts of IL-15Rα for enhanced bioactivity and extended half-life (7–10). Many of these drug developments are being evaluated in clinical studies, usually in combination strategies, e.g., with checkpoint inhibitors, adoptive cell transfer, and vaccination (1, 11).

Another family member, IL-7 is crucial in the development, survival, and homeostasis of naïve and memory T cells (12). In cancer treatment studies, IL-7 boosted CD4+ and CD8+ T cell subpopulations but not Treg expansion and increased TCR repertoire diversity (13). Currently, IL-7 is being investigated for adoptive cell transfer (ACT) in the context of ex-vivo cell expansion (14), co-treatment (15), or as part of CAR T cell design (16, 17). The combination of IL-7 and IL-15 was reported to be effective for ex vivo expansion of minimally differentiated T cells (18, 19).

Another member of interest, IL-21, plays an important role in the differentiation of B cells into plasma cells and the development of TH17 cells. Furthermore, it enhances the proliferation of lymphocytes and the cytotoxicity of CD8+ T cells and NK cells, and has a negative effect on Treg production (20). Preclinical studies indicated that IL-21 enhanced the cytotoxicity and persistence of NK cells and CTLs in tumors (21, 22). Interestingly, strong synergistic effects were reported ex vivo when IL-21 was combined with IL-15, promoting the expansion and cytotoxicity of CD8+ T cells and NK cells. In addition, increased antitumor effects were shown in tumor mouse models by ACT or recombinant cytokine treatment (23, 24). Currently, clinical studies with IL-21, as well as IL-15 or IL-7, are mostly early-phase combination studies with checkpoint inhibitors, adoptive cell transfer or vaccination (1).

An important challenge for the cytokine application is dose-limiting systemic toxicity. Antibody-cytokine fusion proteins have emerged as a strategy to address this problem by targeting-mediated cytokine enrichment at the tumor site, i.e., enabling effective local concentration at lower dosage. Antitumor effects have been demonstrated for fusion proteins composed of antibodies targeting different tumor-associated antigens (e.g., CEA, EDB, EDA, GD2, EGFR, FAP) and many cytokines of the common gamma chain receptor family (e.g., IL-2, IL-15, IL-15/IL15Rα, IL-21, IL-7) (25–32). To generate these bifunctional fusion proteins, different antibody formats were used (e.g., IgG, Db, scFv, VHH) and in some cases, cytokine activity was modified. However, the efficacy of monotherapies is limited and combinatorial treatments are required, whereupon the combination of cytokines hold potential (18, 23, 24, 33). Tumor targeted co-delivery of different cytokines in a single trifunctional molecule is expected to synergize their activity in spatial and temporal manner, thereby enhancing their impact. To date, tumor-directed delivery of IL-15/4-1BBL (scFv anti-FAP) (34, 35), IL-2/TNF (scFv anti-EDA) (36) and IL-2/IL-12 (scFv-Fc anti-CD30, LAIR2) (37, 38), has shown significant therapeutic effects in preclinical tumor mouse models. Here, we report the design of novel trifunctional tumor-directed antibody-cytokine fusion protein formats combining IL-15 with IL-21 or IL-7. Analysis of cytokine activity with the fusion proteins either bound to target cells or in solution revealed combinatory benefits of the two-in-one molecule approach, especially in target-bound form. Format-related differences in their immunomodulatory potential are highlighted.

## 2 Materials and methods

### 2.1 Materials

Antibodies and other reagents were purchased from BioLegend (anti-CCR7 PE, 353204; anti-CD3 PE, 317308; anti-CD3 PerCP/Cyanine5.5, 317336; anti-CD45RA APC, 304150; anti-STAT3 Phospho (Tyr705) FITC, 651020; anti-STAT5 Phospho (Tyr694) APC, 936906; FoxP3 Fix/Perm buffer, 421401; FoxP3 Perm buffer, 421402), and Miltenyi Biotech (anti-His-PE, 130-120-718; anti-CD4 VioBlue, 130-114-534; anti-CD8 PEVio770, 130-110-680; anti-CD8 VioBlue, 130-110-683; anti-Granzyme B FITC, 130-101-355; CytoStim™ (130–092–173). Human IFN-γ (DY285) DuoSet^®^ ELISA kit was purchased from R&D Systems. B16-FAP cells (transfectants with human FAP) (K. Pfizenmaier, University of Stuttgart) were cultured in RPMI 1640 (Life Technologies, 11875), 5% FBS (PAN Biotech, P30-3309), supplemented with 200 µg/ml zeocin (Thermo Fisher Scientific, J67140.XF). HEK293-6E cells were cultured in Freestyle F17-Medium (Thermo Fisher Scientific, A1383501) supplemented with 4 mM GlutaMAX-I (Thermo Fisher Scientific, 35050038), 0,1% Kolliphore P188 (Merck, K4894). Human peripheral blood mononuclear cells (PBMC) were isolated from buffy coat of healthy donors (Klinikum Stuttgart/Institut für Klinische Transfusionsmedizin und Immungenetik Ulm gemeinnützige GmbH, Germany) by Ficoll density gradient centrifugation (Lymphocyte Separation Medium 1077, Promocell, C-44010) and cultivated in RPMI 1640, 10% FBS. CellTrace™ CFSE Proliferation kit (C34554) was purchased from Thermo Fisher Scientific and Mitomycin C (M0503) from Merck.

### 2.2 Generation of antibody-cytokine fusion proteins

**Figure 1:**
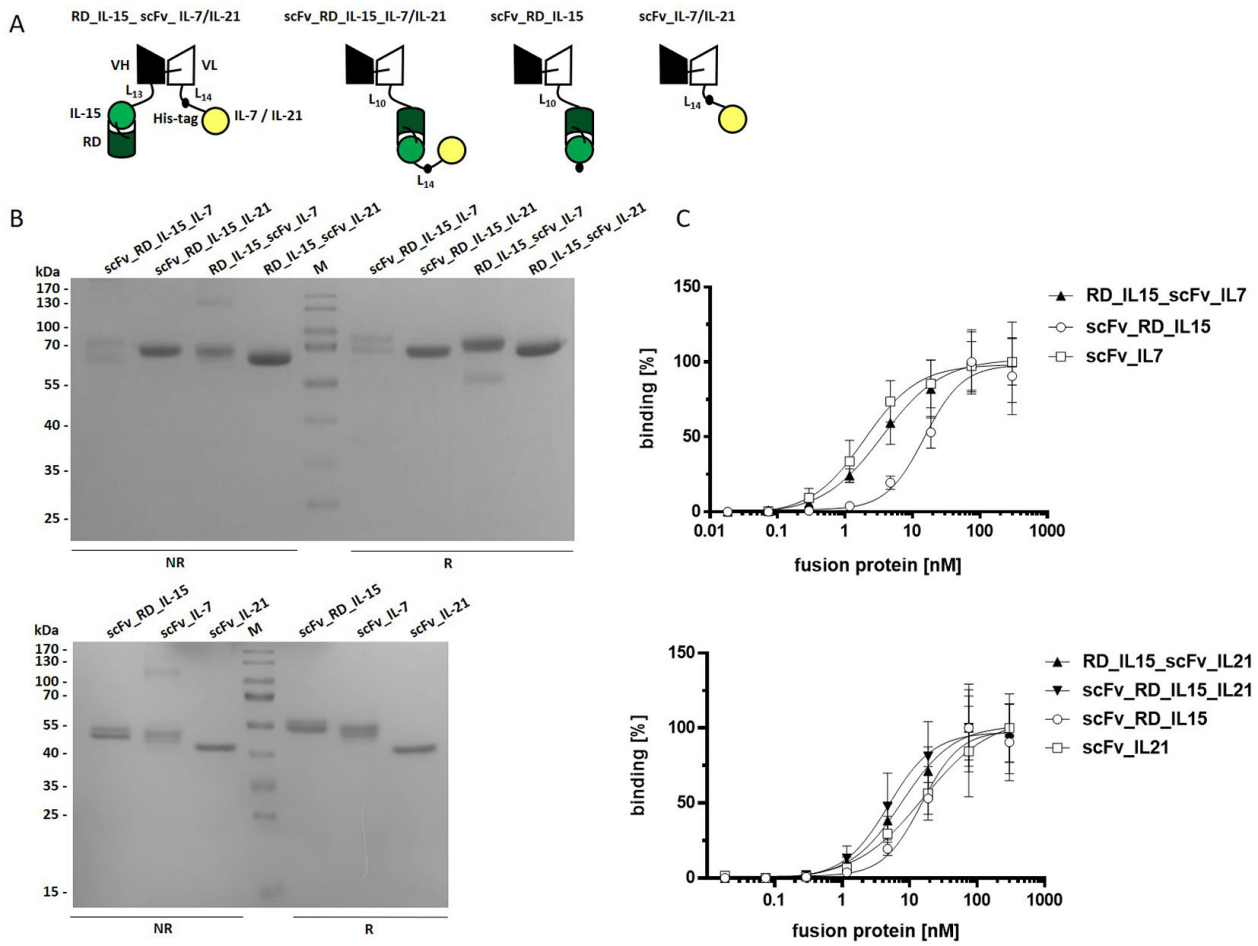
Trifunctional scFv-based fusion protein variants combining RD_IL-15 with IL-7 or IL-21. **(A)** Schema of the recombinant proteins. V_H/L_, variable region of the heavy/light antibody chain; RD, receptor domain of IL15Rα (aa 31-107); black dot, hexahistidyl-tag; Ln, linker with n amino acids **(B)** 12% SDS-PAGE analysis of fusion proteins (2 µg/lane) under non reducing (NR) and reducing (R) conditions. Coomassie staining. **(C)** Binding analysis of fusion proteins to B16-FAP cells by flow cytometry. Bound fusion protein was detected by anti-hexahistidyl-tag-PE antibody. Graphics show mean ± SD, n=3.

FAP-directed bifunctional scFv antibody fusion proteins were cloned based on scFvmo36_RD_IL-15 (25). The C-terminally located cytokine component was replaced by human IL-7 (UniProtKB P13232) and human IL-21 (UniProtKB Q9HBE4), respectively, generating scFv_IL-7/IL-21. Trifunctional scFv antibody fusion proteins with cytokines arranged in parallel, e.g., with the antibody in between, were cloned by fusing the RD_IL-15 moiety to the N-terminus of the corresponding bifunctional scFv antibody fusion proteins with IL-7 and IL-21, leading to RD_IL-15_scFv_IL-7/IL-21. Trifunctional scFv antibody fusion proteins with cytokines arranged in series, e.g., connected to each other, were generated by fusing IL-7 or IL-21 at the C-terminus of scFvmo36_RD_IL-15, resulting in scFv_RD_IL-15_IL-7/IL-21. A hexahistidyl-tag was introduced (see **Figure 1A**). Fab antibody fusion proteins were cloned based on IgGhu36 (39). RD_IL-15 was fused to the C-terminus of the CH1 domain, and IL-21 was fused to the C-terminus of the CL domain. In addition, a hexahistidyl-tag was introduced C-terminally at the heavy chain. In the constructs, glycine/serine-based linker connecting the antibody fragment with the cytokine component ranged between 10 and 14 amino acids (**Supplementary Table 1**).

Recombinant proteins were produced by transient transfection in HEK-293-6E cells (NRC Biotechnology Research Institute, Canada) according to the standard protocol of the cell line provider. In brief, 200 µg plasmid DNA and 400 µg Polyethylenimine (PEI) (Polysciences, 23966-1) suspended in 10 ml Freestyle F17-medium each were mixed and incubated for 15 min at RT, before addition to 200 ml of HEK293-6E cell suspension (2x10^6^ cells/ml). After 24 h cultivation 5 ml of 20% (w/v) Tryptone N1 (Organotechnie, 19553) was added and cells incubated for additional 72 h. Recombinant protein was purified from the supernatant via immobilized metal ion affinity chromatography (IMAC) (40) and if required, size-exclusion chromatography (Superdex™200 increase, 10/300 GL, GE Healthcare).

### 2.3 Thermostability assay

Thermostability of the fusion proteins was determined by dynamic light scattering with the ZetaSizer Nano ZS (Malvern). Purified protein in PBS (0.1 mg/ml) was sterile-filtered and exposed to increasing temperatures (35°C to 85°C) in 1°C intervals with 2-minute equilibration steps. The melting point was defined by the starting point of the increase in light scattering.

### 2.4 Binding analysis

For flow cytometry analysis, 2x10^5^ target cells/well were incubated with the respective fusion protein for 1 h at 4°C in U-bottom 96-well-plates. After washing, bound protein was detected by PE-conjugated anti-hexahistidyl-tag antibody (1 h, 4°C), respectively. After washing, fluorescence was measured by MACSQuant Analyzer10/VYB (Miltenyi Biotech), and data were analyzed using FlowJo (Tree Star). Relative mean fluorescence intensity (MFI) was calculated as (MFI_sample_-(MFI_detection system_-MFI_cells_))/MFI_cells_.

### 2.5 STAT3/STAT5 phosphorylation analysis

In the targeted setting, 2x10^4^ B16-FAP cells/well were seeded in F-bottom 96-well-plates. In parallel, PBMCs were thawed. The next day, cells were washed and incubated with 10 nM fusion protein for 1 h at room temperature (RT). After washing (0,5% BSA in PBS), 2x10^5^ PBMCs were added, and plates were shortly centrifuged followed by 15 min incubation at 37°C. In the untargeted setting, PBMCs were incubated directly with the fusion protein for 15 min at 37°C. Next, fixation buffer was added (on ice, 5 min). Cells were washed (0,5% BSA in PBS), and precooled True-Phos Perm Buffer was added (15 min, -20°C). Finally, cells were washed, stained with anti-STAT5-APC and anti-STAT3-FITC (30 min, RT), washed again and analyzed by MACSQuant Analyzer10 (Miltenyi Biotech).

### 2.6 Proliferation assays

2x10^4^ B16-FAP cells/well were seeded in F-bottom 96-well-plates. In parallel, PBMCs were thawed. The next day, PBMCs were labeled for 15 min at 37°C with CFSE (625 nM, 10^6^ cells/ml) followed by 5 min incubation on ice. In the targeted setting, B16-FAP cells were arrested by 2 h incubation at 37°C with mitomycin (10 µg/ml). Cells were washed and incubated with the fusion proteins for 1 h at 37°C. After removing the unbound fusion protein by washing, CytoStim™ (suboptimal concentration) and 2x10^5^ PBMC/well were added. In the untargeted setting, PBMCs were directly incubated with the fusion proteins. After 6 days culture, T cells were stained with fluorescence-conjugated antibodies targeting corresponding subpopulation markers (1h, 4°C), and cell proliferation was measured by flow cytometry (MACSQuant Analyzer10/VYB). CD4+ and CD8+ T cell (CD3+) subpopulations: naïve (CCR7+CD45RA+); EM, effector memory (CCR7-CD45RA-); E, effector cells (CCR7-CD45RA+). Data were analyzed using FlowJo (Tree Star).

### 2.7 IFN-γ release assay

2x10^4^ B16-FAP cells/well were seeded in F-bottom 96-well-plates. In parallel, PBMCs were thawed. The next day, B16-FAP cells were arrested with mitomycin (2 h, 37°C), followed by washing and the incubation with the fusion proteins (1 h, 37°C or RT ). After washing, CytoStim™ (suboptimal concentration) and 2x10^5^ PBMC/well were added. In the untargeted setting, PBMCs were directly incubated with the fusion proteins. After 2 days or 5 days of culture, the supernatant was harvested, and IFN-γ concentration was determined via sandwich ELISA (DuoSet ELISA kit) according to the manufacturer’s instructions.

### 2.8 Granzyme B expression assay

2x10^4^ B16-FAP cells/well were seeded in F-bottom 96-well-plates. In parallel, PBMCs were thawed. The next day, B16-FAP cells were arrested with mitomycin (2 h, 37°C), followed by washing and the incubation with the fusion proteins (1 h, RT). After washing, CytoStim™ (suboptimal concentration) and 2x10^5^ PBMC/well were added. In the untargeted setting, PBMCs were directly incubated with the fusion proteins. After 7 days of culture, cells were fixed (20 min, RT, FoxP3 Fix/Perm buffer, permeabilized (15 min, RT, FoxP3 Perm buffer), and stained with anti- CD3 PE, anti-CD8 VioBlue, and anti-Granzyme B FITC antibodies (1 h, 4 °C). Cells were washed and measured by flow cytometry (MACSQuant Analyzer10/VYB), and data were analyzed using FlowJo (Tree Star).

### 2.9 Statistical analysis

Unless otherwise stated, all data are represented as mean ± S.D. of three independent experiments. Block shift correction was performed according to the formula: X’_n_ = X_n_ – (Y_n_ – Y), with X’_n_ being the corrected value of X from the experiment n, Y the average of the X values from all experiments performed, and Y_n_ the average of the duplicate values of X from experiment n. Statistical significance was determined using one-way ANOVA followed by Tukey’s post-test (Graphpad Prism). *P* values below 0.05 were considered statistically significant (*** *P* < 0.001, ** *P* < 0.01, * *P* < 0.05).

## 3 Results

We generated trifunctional antibody-cytokine fusion proteins composed of a scFv antibody directed against the fibroblast activation protein (FAP) as a tumor associated model antigen, IL-15 fused to an IL-15Rα fragment (RD), and either IL-7 or IL-21. The molecules were generated in two configurations: i) the cytokines were fused to the N-terminus and C-terminus of the scFv, respectively (RD_IL-15_scFv_IL-7/IL-21), and ii) the cytokines were fused in series and linked to the C-terminus of the scFv (scFv_RD_IL-15_IL-7/IL-21). In addition, bifunctional molecules, fusing the respective cytokines to the C-terminus of the scFv (scFv_RD_IL-15/IL-7/IL-21) were produced (see **Figure 1A**). The fusion proteins were transiently transfected and expressed in HEK293-6E cells and purified via IMAC. Average yields of fusion proteins with IL-21 (4 - 9 mg/L) were significantly higher than those of most fusion proteins containing IL-7 and that with IL-15 only (2 mg/L). scFv_RD_IL-15_IL-7 was expressed at low yields (0.2 mg/L) and was therefore excluded from further assays. Analysis by SDS-PAGE showed bands of the expected sizes (bifunctionals 44-51 kDa, trifunctionals 67-69 kDa) (**Supplementary Table 1**), with a broad band pattern consistent with cytokine glycosylation (**Supplementary Image 1**). RD_IL-15_scFv_IL-7 exhibited an additional faint band under reducing conditions, indicating the presence of a minor cleavage product (**Figure 1B**). Binding analysis to B16-FAP cells confirmed concentration-dependent binding with EC_50_ values in the low nanomolar range (**Figure 1C**, **Supplementary Table 2**).

### 3.1 Trifunctional antibody (scFv)-fusion proteins combining RD_IL-15 with IL-7

**Figure 2:**
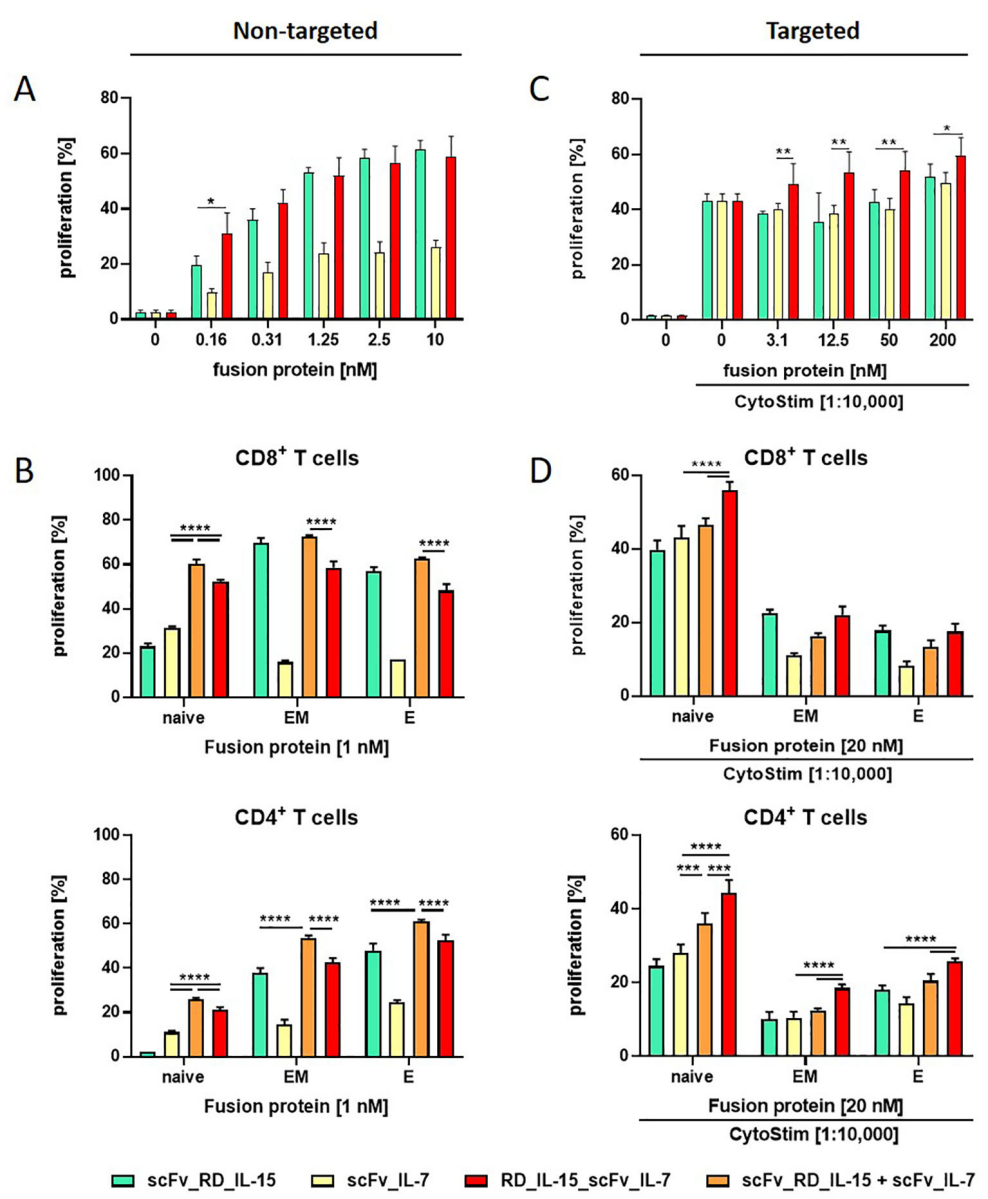
The proliferation effect of RD_IL-15_scFv_IL-7 depends on the T cell subtype. Effect of untargeted **(A, B)** and B16-FAP cell targeted **(C, D)** fusion proteins on the proliferation of PBMCs **(A, C)** and CD4+ or CD8+ T cell subpopulations (naïve (CCR7+CD45RA+); EM, effector memory (CCR7-CD45RA-); E, effector cells (CCR7-CD45RA+)) **(B, D)**. CFSE-labeled PBMCs were incubated with the fusion proteins either in solution (0.16-10 nM/1nM) or targeted to B16-FAP cells (preincubated at 3.1-200 nM/20 nM and then washed to remove unbound fusion protein) in presence of suboptimal concentration of CytoStim™. After 6 days, multicolor antibody staining of CD3, CD8, CD4, CD45RA and CCR7 was conducted and proliferation of subpopulations analyzed by flow cytometry. Graphics show mean ± SD, n=3 with blockshift correction **(B-D)**, *P values, *p<0.05; **p<0.01, ***p<0.001; ****p<0.0001.

The cytokine activity of the bi- and trifunctional antibody fusion proteins was assessed in a model system by stimulating PBMC proliferation in untargeted and targeted form. In the untargeted setting, resting PBMCs were incubated with the fusion proteins in solution. In the targeted setting, PBMCs were activated with a suboptimal concentration of CytoStim™ (T cell superagonist) and incubated with the fusion proteins bound to B16-FAP cells, after washing away unbound fusion protein. In solution, all fusion proteins were active, with the trifunctional RD_IL-15_scFv_IL-7 being similarly effective as scFv_RD_IL-15 and both clearly more efficient than scFv_IL-7 (**Figure 2A**). Subpopulation analysis of CD8+ and CD4+ T cells confirmed this pattern for effector memory and effector cells, underscoring the dominant role of IL-15 for these subpopulations. For naïve cells, the individual effects of IL-15 and IL-7 were more alike, with IL-7 showing a slight advantage. A clear enhancement effect (1.7-fold CD8+T cells, 1.9-fold CD4+ T cells) was observed for the trifunctional fusion protein and an even greater effect seen for the combination of bifunctional fusion proteins (1.9-fold CD8+T cells, 2.4-fold CD4+ T cells), indicating that naïve T cell subpopulations are particularly responsive to the IL-7/IL-15 combination (**Figure 2B**). In cell-bound form, the effects of bi- and trifunctional fusion proteins further enhancing the proliferation of activated PBMCs were only minor. However, RD_IL-15_scFv_IL-7 tended to be more effective than the individual bifunctional fusion proteins (**Figure 2C**). Nevertheless, also in this setting, subpopulation analysis demonstrated that naïve T cells exhibited a superior response to the trifunctional fusion protein compared to either the individual (1.3-fold CD8+T cells, 1.6-fold CD4+ T cells) or the combined (1.2-fold CD8+ or CD4+ T cells) bifunctional fusion proteins. Furthermore, the effect of the trifunctional fusion protein on effector memory and effector cells was equivalent to that of the dominant bifunctional scFv_RD_IL-15 on CD8+ T cells and slightly enhanced on CD4+ T cells (**Figure 2D**). Thus, the activity of the trifunctional fusion protein was confirmed in targeted form, and its combinatorial potential also demonstrated on activated T cells. Additional analysis of effector functions, including IFN-γ release and granzyme B expression, demonstrated activity, but did not reveal any combinatorial advantage for RD_IL-15_scFv_IL-7 (data not shown). In summary, the trifunctional fusion protein RD_IL-15_scFv_IL-7 exhibited strong activity, matching at the least the effect of the dominant bifunctional scFv_RD_IL-15 fusion protein. Its cooperative activity enhanced the proliferation of additional, mainly naïve T cell subsets, thereby expanding the immune response.

### 3.2 Trifunctional antibody (scFv)-fusion proteins combining RD_IL-15 with IL-21

**Figure 3:**
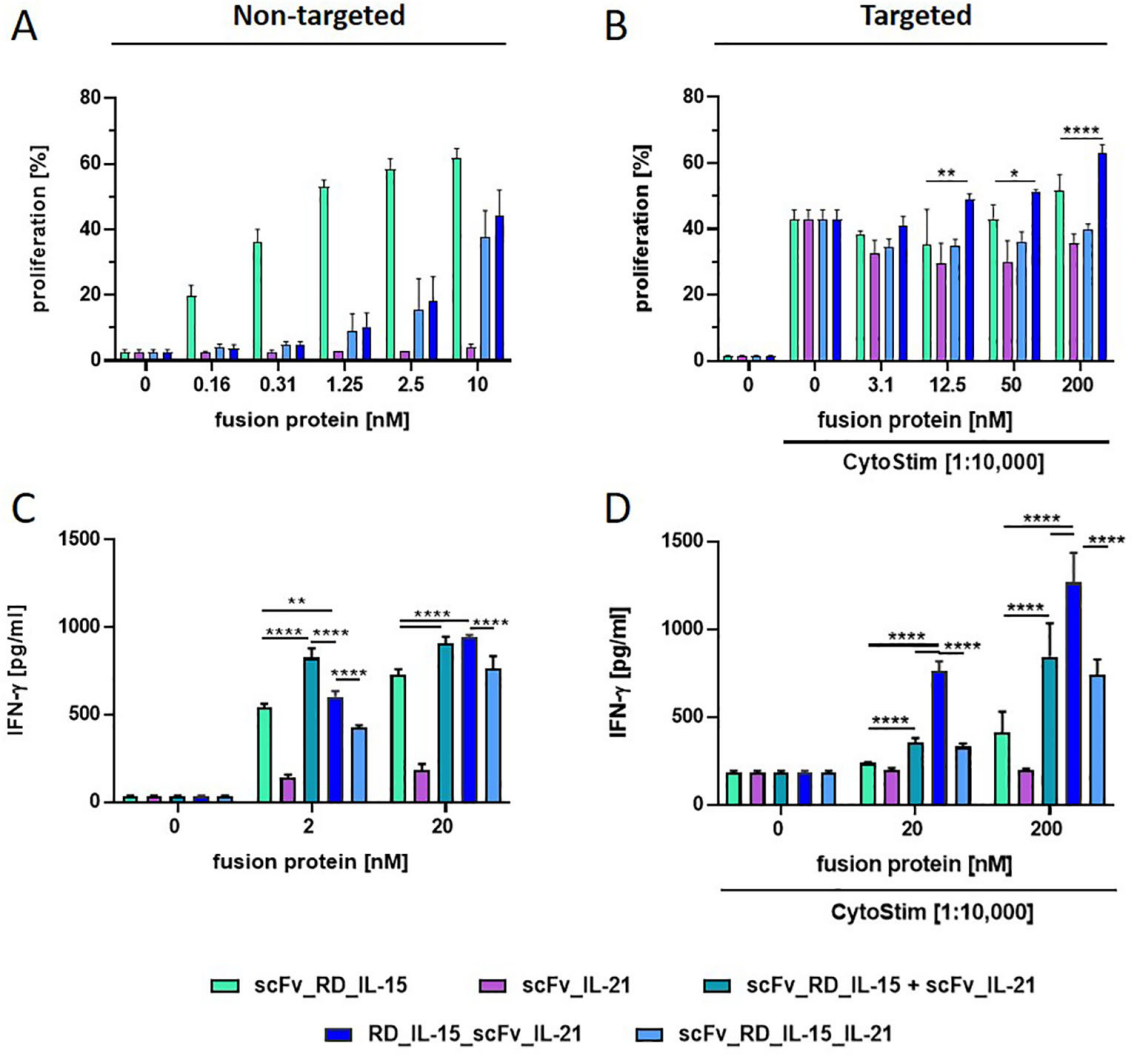
Comparison of scFv-based (RD_IL-15 + IL-21) fusion protein variants in stimulating PBMC proliferation and IFN-γ release. Fusion proteins were incubated (1 h at 37°C) untargeted (0.16-10 nM/2 and 20 nM) **(A, C)** or targeted to B16-FAP cells (preincubated with 3.1-200 nM/20 and 200 nM and then washed to remove unbound fusion protein) in presence of suboptimal concentration of CytoStim™ **(B, D)** with PBMCs. Proliferation of CFSE-labeled PBMCs was measured after 6 days by flow cytometry **(A, B)**. IFN-γ release into the supernatant was determined after 5 days by sandwich ELISA **(C, D)**. Graphics show mean ± SD, n=3, with blockshift correction **(B-D)**, *p<0.05; **p<0.01; ****p<0.0001.

According to previous reports, IL-21 was not expected to induce the proliferation of T and NK cells by itself, but to cooperate with IL-15 in their expansion (23). Indeed, unlike scFv_RD_IL-15, scFv_IL-21, did not induce significant proliferation of resting PBMCs in solution. Unexpectedly, the activities of the untargeted trifunctional RD_IL-15_scFv_IL-21 and scFv_RD_IL-15_IL-21 were not enhanced, but reduced by approximately 16-fold compared to scFv_RD_IL-15, suggesting a rather masking role for IL-21 (**Figure 3A**). However, when targeted to B16-FAP cells, activity was regained, and RD_IL-15_scFv_IL-21 but not scFv_RD_IL-15_IL-21, was slightly more effective than scFv_RD_IL-15 in enhancing the proliferation of activated PBMCs (**Figure 3B**). To further assess the combinatorial potential in a different readout, IFN-γ release was analyzed. In this context, the bifunctional scFv_IL-21 alone was already effective in solution, albeit to a lesser extent than scFv_RD_IL-15. Combining both bifunctional fusion proteins (untargeted or targeted) led to a clear enhancement effect (up to 1.6 and 3-fold). In solution, the trifunctional RD_IL-15_scFv_IL-21, but not scFv_RD_IL-15_IL-21, also showed cooperative activity, that was similar effective at high concentration but less efficient at low concentration compared to the combination of bifunctional fusion proteins (**Figure 3C**). Interestingly, in targeted form, the cooperative effect of RD_IL-15_scFv_IL-21 was clearly superior and strongly synergistic (4.7 and 10.5-fold) (**Figure 3D**). Targeted scFv_RD_IL-15_IL-21 also showed cooperative activity, but the effect was less pronounced and only similar to that of the combined bifunctional fusion proteins. Thus, the colocalized cell surface presentation of the cytokines by the RD_IL-15_scFv_IL-21 format proved clearly advantageous, leading to strong cooperative effects in terms of IFN-release and furthermore showing support for the proliferation of activated PBMCs. The more restricted activity of the trifunctional fusion protein in solution might be considered favorable, as it is expected to reduce systemic effects, thus further supporting the concept of a targeted strategy.

### 3.3 Trifunctional antibody (Fab)-fusion proteins combining RD_IL-15 with IL-21

**Figure 4:**
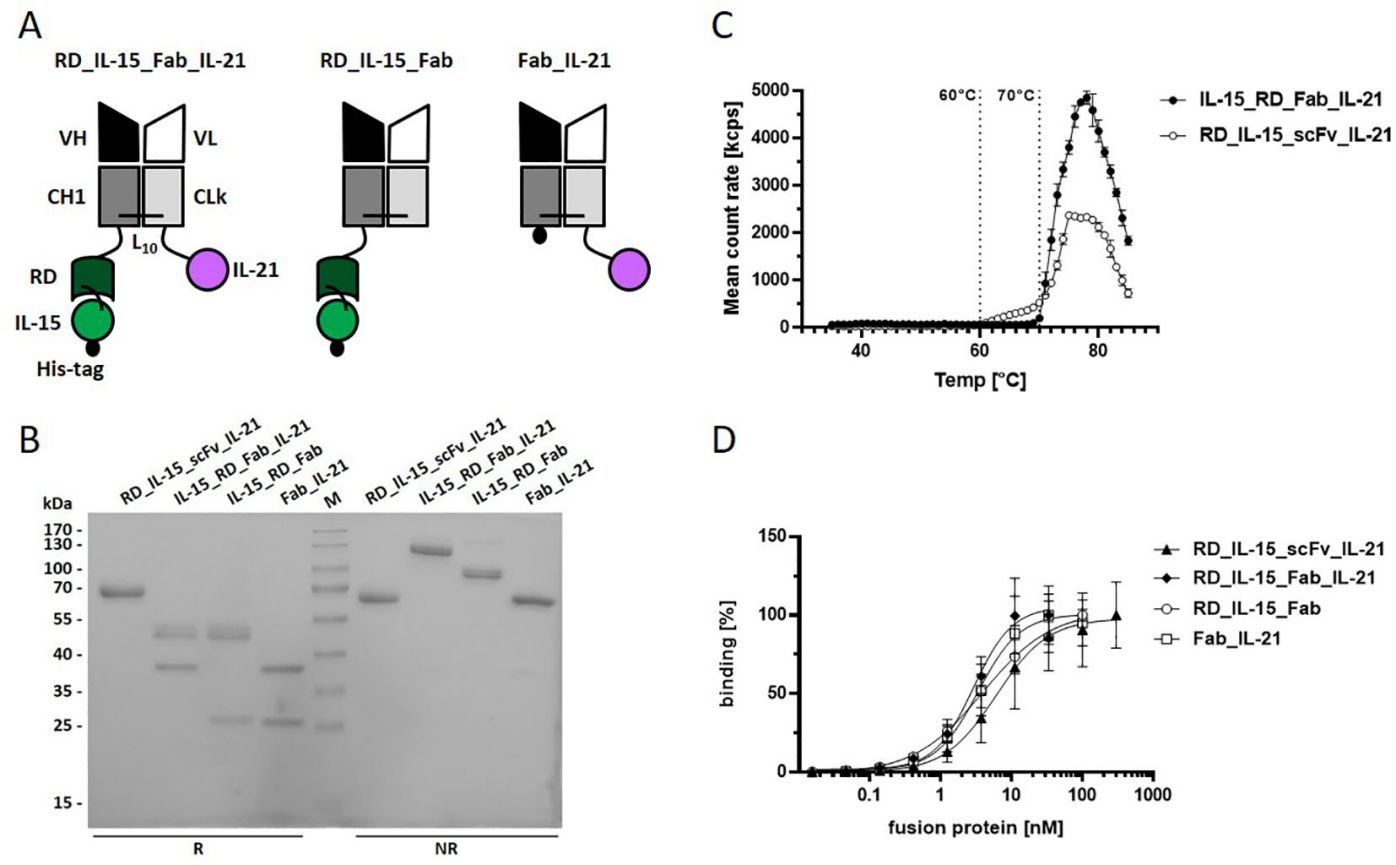
Trifunctional Fab-based fusion protein variant combining IL-15_RD with IL-21. **(A)** Schema of the recombinant proteins. V_H/L_, variable region of the heavy/light antibody chain; CH1, constant domain 1 of γ1 heavy chain; CLk, constant domain of kappa light chain; RD, receptor domain of IL15Rα (aa 31-107); black dot, hexahistidyl-tag; Ln, linker with n amino acids **(B)** 12% SDS-PAGE analysis of fusion proteins (3 µg/lane) under reducing (R) and non-reducing (NR) conditions. Coomassie staining. **(C)** Thermostability analysis of the fusion proteins (0.1 mg/ml) by dynamic light scattering with the ZetaSizer Nano ZS (Malvern). **(D)** Binding analysis of fusion proteins to B16-FAP cells by flow cytometry. Bound fusion protein was detected by anti-hexahistidyl-tag-PE antibody. Graphics show mean ± SD, n=3.

To validate the concept and further improve the molecular design, we generated another novel trifunctional antibody-cytokine fusion protein format, using Fab as the antibody component. In this construct, RD_IL-15 and IL-21 were fused to the C-terminus of the constant domains CH1 and CL from the Fab chains, respectively (**Figure 4A**). By introducing this larger compact antibody fragment, we expected to enhance stability and improve targeted cytokine presentation and activity. IL-15_RD_Fab_IL-21 and the corresponding bifunctional controls IL-15_RD_Fab and Fab_IL-21 were produced with comparable yields to their scFv-fusion protein equivalents. SDS-PAGE analysis confirmed correct chain expression and dimerization (**Figure 4B**). Analysis of thermostability by dynamic light scattering showed a higher melting point, i.e., stability, for IL-15_RD_Fab_IL-21 (70°C) compared to RD_IL-15_scFv_IL-21 (60°C) (**Figure 4C**). Similar target-binding capacity of the Fab-fusion proteins was confirmed by flow cytometry (**Figure 4D**).

**Figure 5:**
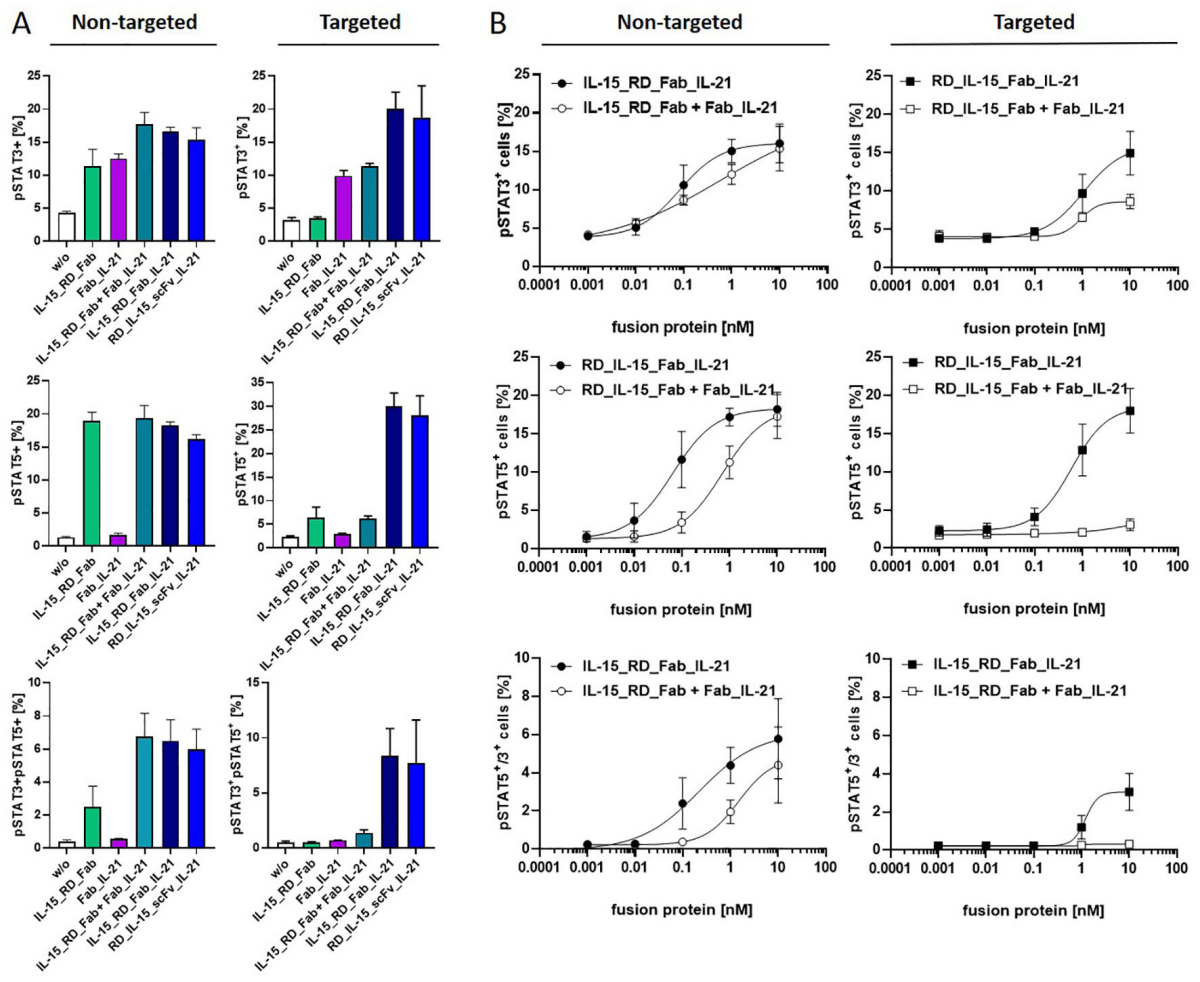
Cooperative receptor activation by IL-15_RD_Fab_IL-21 enhances JAK-STAT pathway signaling. Fusion proteins were incubated untargeted or targeted to B16-FAP cells at saturating concentration (10 nM) **(A)** or titrated (0.001-10 nM) **(B)** with PBMCs for 15 minutes at 37°C. Cells were fixed, pSTAT3 and pSTAT5 intracellularly stained, and analyzed by flow cytometry. Graphics show mean ± SD, n=2 **(A)**, n=3 **(B)**.

To confirm cytokine activity and gain further insight into combined receptor stimulation, cytokine receptor activation on PBMCs was assessed by measuring JAK-STAT pathway activation via STAT3 and STAT5 phosphorylation. IL-15 and IL-21 have been previously described to primarily activate STAT5 and STAT3, respectively, illustrating differences in the pathway signaling (41). This pattern was corroborated with Fab_IL-21 inducing pSTAT3, but not pSTAT5, while IL-15_RD_Fab effectively induced pSTAT5 and, to lesser extent, pSTAT3 (**Figure 5A**). At saturating concentration, the combination of bifunctional fusion proteins in solution led to a clear increase in pSTAT3+ and double-positive pSTAT3+/pSTAT5+ cells, indicating cooperative receptor activation. Similar effects were obtained with the untargeted trifunctional fusion proteins. In contrast, bound to target cells strong cooperative pathway signaling was achieved by the trifunctional, but not by the combined bifunctional fusion proteins (**Figure 5A**). In terms of signaling dynamics, the untargeted trifunctional molecule at a subsaturating concentration demonstrated superior efficacy during the early phase of signaling, exhibiting an enhanced peak at 30 minutes for pSTAT3+ and double-positive pSTAT3+pSTAT5+ cells, followed by a rapid decline, at which point the efficacy of the trifunctional and combined bifunctional molecules tended to align. In contrast, pSTAT5+ cells showed an early increase and maintained higher levels for up to 2 hours with the trifunctional molecule compared to the combined bifunctional molecules, which took longer to reach their maximum (**Supplementary Image 2A**). Similar dynamics were observed for the fusion proteins in their targeted forms (**Supplementary Image 2B**). Titration of fusion proteins in solution revealed an approximately 7-fold (pSTAT3+ cells, pSTAT3+pSTAT5+ cells) and 12-fold (pSTAT5+ cells) higher potency of the trifunctional IL-15_RD_Fab_IL-21 compared to the combination of bifunctional IL-15_RD_Fab and Fab_IL-21. In targeted form, the efficacy of the trifunctional fusion protein was clearly superior to the combination of bifunctional fusion proteins, enhancing the maximal percentage of pSTAT3+ by 1.7-fold and pSTAT5+ cells by 5.9-fold. Furthermore, induction of pSTAT3+pSTAT5+ cells was only achieved by the trifunctional fusion protein (**Figure 5B**). Thus, combining both cytokines in a single IL-15_RD_Fab_IL-21 molecule, enforcing colocalization, clearly enhanced the potential of cooperative receptor activation, especially in targeted form.

**Figure 6:**
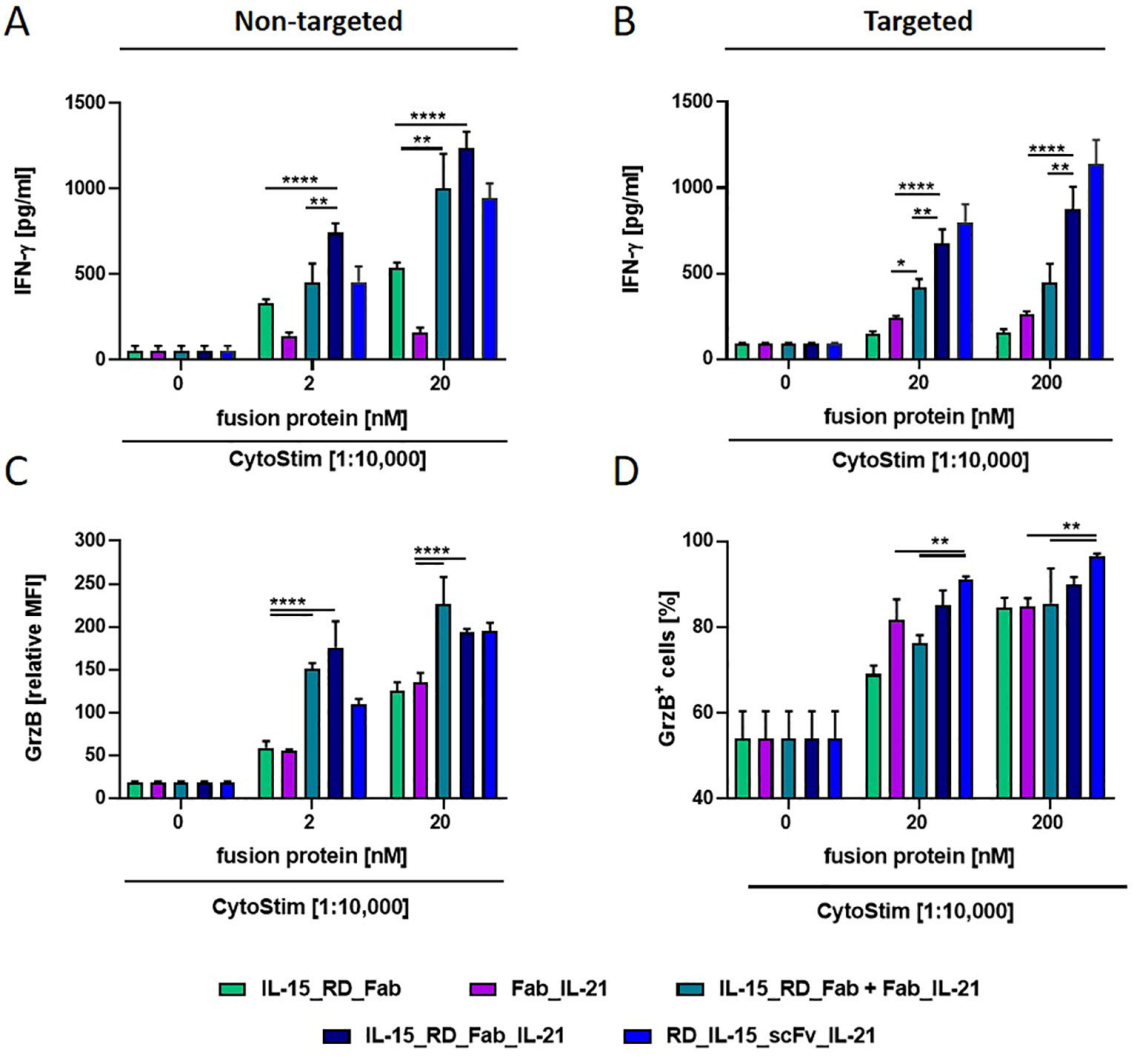
Combinatory potential of IL-15_RD_Fab_IL-21 enhancing IFN-γ release and Granzyme B expression in CD8+ T cells. Fusion proteins were incubated (1 h, RT) either untargeted (2 and 20 nM) **(A, C)** or targeted to B16-FAP cells (preincubated with 20 and 200 nM and then washed to remove unbound fusion protein) **(B, D)**, with PBMCs in presence of suboptimal concentration of CytoStim™. IFN-γ release into the supernatant was determined after 2 days by sandwich ELISA **(A, B)**. Granzyme B expression in CD8+ T cells **(C)** or percentage of Granzyme B expressing CD8+ T cells **(D)** was measured after 7 days by flow cytometry. Graphics show mean ± SD, n=3, with blockshift correction, *p<0.05; **p<0.01; ****p<0.0001.

Next, the immune stimulatory activity of IL-15_RD_Fab_IL-21 was assessed on activated PBMCs by measuring IFN-γ release. Here, IL-15_RD_Fab_IL-21 was significantly more effective than the respective individual (up to 2.5-fold untargeted, 4.6-fold targeted) and combined (up to 1.7 untargeted, 2.2-fold targeted) bifunctional fusion proteins (**Figures 6A, B**). Thus, the combinatorial potential of the trifunctional fusion protein enhancing IFN-γ release was corroborated for the Fab-format.

In addition, we analyzed the cytokine activity of the fusion proteins on the cytotoxic potential of activated CD8+ T cells by measuring granzyme B expression. Untargeted, IL-15_RD_Fab and Fab_IL-21 were similar effective in enhancing granzyme B expression in CD8+ T cells, and these effects could be further increased to a similar degree by their combination or by IL-15_RD_Fab_IL-21 (approx. up to 3.6-fold) (**Figure 6C**). In targeted form, both cytokines had a strong individual effect, while no clear combinatorial benefit was observed (**Figure 6D**). Altogether, the trifunctional IL-15_RD_Fab_IL-21 was highly effective in enhancing IFN-γ release and granzyme B expression, demonstrating the combinatorial potential of this approach in reinforcing immune effector functions. Interestingly, for both read-outs, the activity of IL-15_RD_Fab_IL-21 was comparatively stronger in solution (up to 1.7-fold) and weaker in target-bound form (up to 1.3-fold) than the activity of RD_IL-15_scFv_IL-21. Thus, RD_IL-15_scFv_IL-21 appears to be the more promising format for translating this concept.

## 4 Discussion

The design of trifunctional antibody-cytokine fusion proteins influenced the combined cytokine activity in targeted and untargeted form. Fusing the scFv with the cytokines linked in series resulted in significantly reduced expression rates (scFv_RD_IL-15_IL7/IL-21) and diminished fusion protein activity (scFv_RD_IL-15_IL-21). In this arrangement, IL-21 appeared to partially mask the activity of IL-15, which is placed in the central position of the fusion protein. Although scFv_RD_IL-15_IL-21 still exhibited some capacity to deliver combined cytokine activity in targeted form, enhancing IFN-γ release, the overall immune stimulatory potential of this format seemed limited. Conversely, flanking the scFv at the N- and C-termini with the cytokines resulted in a more favorable arrangement for their presentation on the cell surface, translating into strong cooperative effects in targeted form. Interestingly, in solution, the combinatorial benefit was markedly reduced or abrogated, suggesting impediments to joint cytokine presentation in cis. This indicates that for this format, targeted localized cooperative activity might be favored while systemic activity is reduced. Comparing RD_IL-15_scFv_IL-21 with IL-15_RD_Fab_IL-21 showed that increased stability, shorter linker length, and the more physiological orientation of IL-15_RD in the Fab-format translated into stronger activity in solution but reduced activity in target-bound form. Thus, the enhanced flexibility and shorter cell distancing attributed to the scFv-format seemed overall more advantageous for the trifunctional antibody-cytokine fusion protein approach.

Individually, IL-15 and IL-21 have the potential to stimulate the expansion and effector functions of antigen-specific CD8+T cell and NK cells, mediating antitumor effects (1). In the trifunctional fusion protein, cooperative activity of IL-15 and IL-21 was primarily demonstrated by enhancing IFN-γ release and granzyme B expression of CD8+ T cells, that was influenced by the format and the targeted or untargeted state of the fusion protein. Although the specific combinatorial responses varied, the stimulatory activity of the trifunctional fusion proteins with the antibody in the central position was consistently at least comparable to that of the most potent cytokine. In targeted form, this also extended to the effect on T cell proliferation. The synergistic enhancement of expansion and effector functions in mouse and human CD8+ T cells has been predominantly reported for the combination of wildtype IL-15 and IL-21 at equal ratios, although effective ratios differing by up to 10-fold have also been demonstrated (23). In our trifunctional fusion protein design, the cytokine ratio is fixed at 1:1, where the activity of IL-15 is increased over 10-fold by combining it with the IL-15Rα fragment (25, 42). Signaling bias by asymmetric cross-talk is likely to influence the combined activity. IL-15/IL-21 signaling involves JAK-STAT3/5, PI3K-AKT and MAPK pathways contributing to the cellular responses (41, 43). IL-15 and IL-21 differ in activating primarily STAT5 and STAT3, respectively (41). Our JAK-STAT3/5 pathway activation analysis demonstrated not only the strong effect of IL-15_RD on STAT5 but also cooperative activity with IL-21 on STAT3. The potency and efficacy of the trifunctional fusion protein were notably superior to the combination of the corresponding bifunctional fusion proteins, highlighting the importance of cytokine proximity in the two-in-one molecule approach (**Figure 5**). The availability of the shared common gamma chain (γ_c_) and affinity-mediated preassembly of receptor complexes are critical factors influencing concomitant pathway activation by γ_c_ cytokines (e.g. IL-4/IL-7/IL-21) (44). Modelling cell-specific dynamics and regulation of γ_c_ cytokines based on ligand-receptor interactions and endosomal trafficking kinetics enabled predictions of cell-type specific responses to individual and combined cytokines (e.g. IL-2, IL-15, IL-4/IL-7) (45). This could provide further guidance for molecular engineering, especially when applied to cytokine combinations (e.g., IL-15/IL-21) in the context of fusion proteins.

The rational for using the combination of IL-15 with IL-7 in the context of adoptive cell transfer is to promote ex-vivo the expansion of minimally differentiated T cells, as enhancing T cell stemness is associated with increased proliferation, persistence and antitumor activity, although the precise balance of cytokines remains to be defined (18). Our findings that RD_IL-15_scFv_IL-7 showed cooperative activity mainly in enhancing the proliferation of naïve T cells, along with the strong activity of RD_IL-15 on effector memory and effector cells, particularly CD8+ T cells, suggests the generation of a fusion protein with an extended immune stimulatory profile. Previous studies by Song et al. demonstrated that intratumoral treatment with untargeted IL-7_IL-15 fusion protein was superior to the combination of individual cytokines, inducing tumor growth inhibition in various syngeneic tumor mouse models. This was attributed to higher tumor infiltration of CD4+ and CD8+ T cells, DCs, NK and NKT cells, and the reduction of Tregs, suggesting a comprehensive combinatorial effect on the immune response (33). Targeting both cytokines as trifunctional antibody-fusion protein to the tumor should enable local accumulation and retention, reinforcing the antitumor response. Despite the combinatorial benefit, the development of trifunctional fusion proteins may be challenged by low protein expression and the pronounced sink effect of IL-7, compromising on-target delivery (31). Notably, tumor targeting was achieved and shown relevant for the treatment efficacy of bifunctional antibody-fusion proteins with all three cytokines (IL-15, IL-21 and IL-7) in immunocompetent mouse models (25, 28–31). However, biodistribution studies have also shown varying degrees of off-site accumulation of fusion proteins, dependent on cytokine type and influenced by the fusion protein format (28, 31, 46). This may generate safety issues and become particularly relevant for antibody-fusion proteins featuring two different cytokines. Differences in the molecular size of bi- and trifunctional fusion proteins also warrant attention. A key challenge in the design of small-format immunocytokines lies in the fact that smaller size is associated with faster diffusion, which favors rapid tumor accumulation. However, when applied systemically, the faster blood clearance compromises tissue uptake and exposure, thereby reducing the maximal tissue concentration. Local administration can maximize the bioavailability at the tumor site, whereas larger proteins (> 60 kDa) provide the advantage of longer retention and lymphatic drainage (see review (47)). Trifunctional fusion proteins, due to their larger size, may have an advantage over corresponding bifunctional fusion proteins in this context. Therefore, it is interesting that intratumoral application of targeted cytokines with local retention is shown effective and receiving increasing attention (38, 48). Also, further format development could emphasize targeting-dependent cytokine activity through strategies e.g., like masking or mutants, reported effective in immunocytokines with TNF, IFN-γ and IL-2 (49, 50). Positioning the IL-15Rα fragment holds potential to modulate the cytokine activity. Previous studies have shown that untargeted RD_IL-15_scFv was slightly less active than scFv_RD_IL-15, indicating that the position of the IL-15Rα fragment influences the cytokine activity (34). This may account in part for differences observed in the activity of untargeted RD_IL-15_scFv_IL-21 and IL-15_RD_Fab_IL-21, where the positioning of RD resembles this situation (**Figure 6**). So far, the configuration RD_IL-15_scFv has been successfully integrated into trifunctional fusion proteins with costimulatory ligands of the TNF superfamily, generating molecules with strong targeting-mediated cooperative activity (34, 35). In other studies, shortening the linker length between the antibody and the cytokine in an IL-15Rα_IL-15_IgG fusion protein reduced cytokine activity and concomitant dose-limiting toxicity in solution, while antibody-mediated targeting to PD-1 expressing T cells recovered the IL-15 activity in cis (51). Therefore, modulating the dominant IL-15 activity by linker and configuration design could further balance and strengthen the trifunctional antibody-cytokine fusion protein design.

In summary, our results highlight the cooperative potential of IL-15/IL-7 and IL-15/IL-21 in a targeted two-in-one molecule approach, illustrating the impact of design on the immune response and laying the groundwork for further molecular developments.

## Data Availability

The original contributions presented in the study are included in the article/**Supplementary Material**. Further inquiries can be directed to the corresponding author.
