# Supplementary figures and images for: Trifunctional antibody-cytokine fusion protein formats for tumor-targeted combination of IL-15 with IL-7 or IL-21
Source: Front Immunol. 2025 Apr 30;16:1498697. doi: 10.3389/fimmu.2025.1498697 (PMC12075275; doi:10.3389/fimmu.2025.1498697)

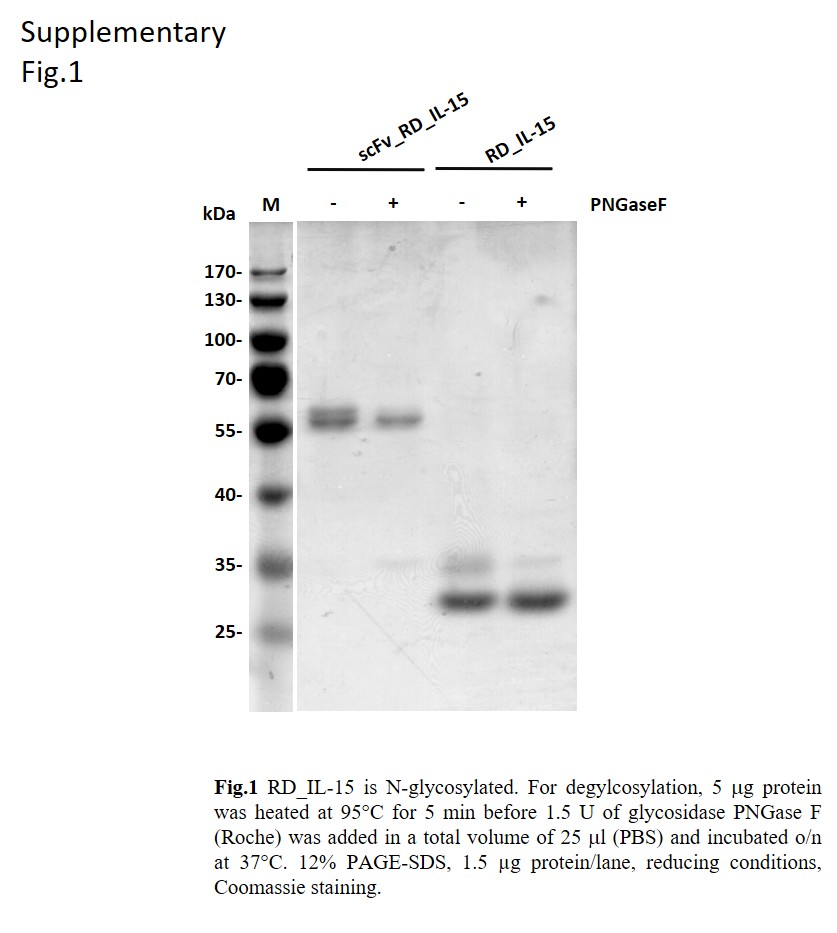

Supplement: Supplementary file 1 [file Image1.jpeg]

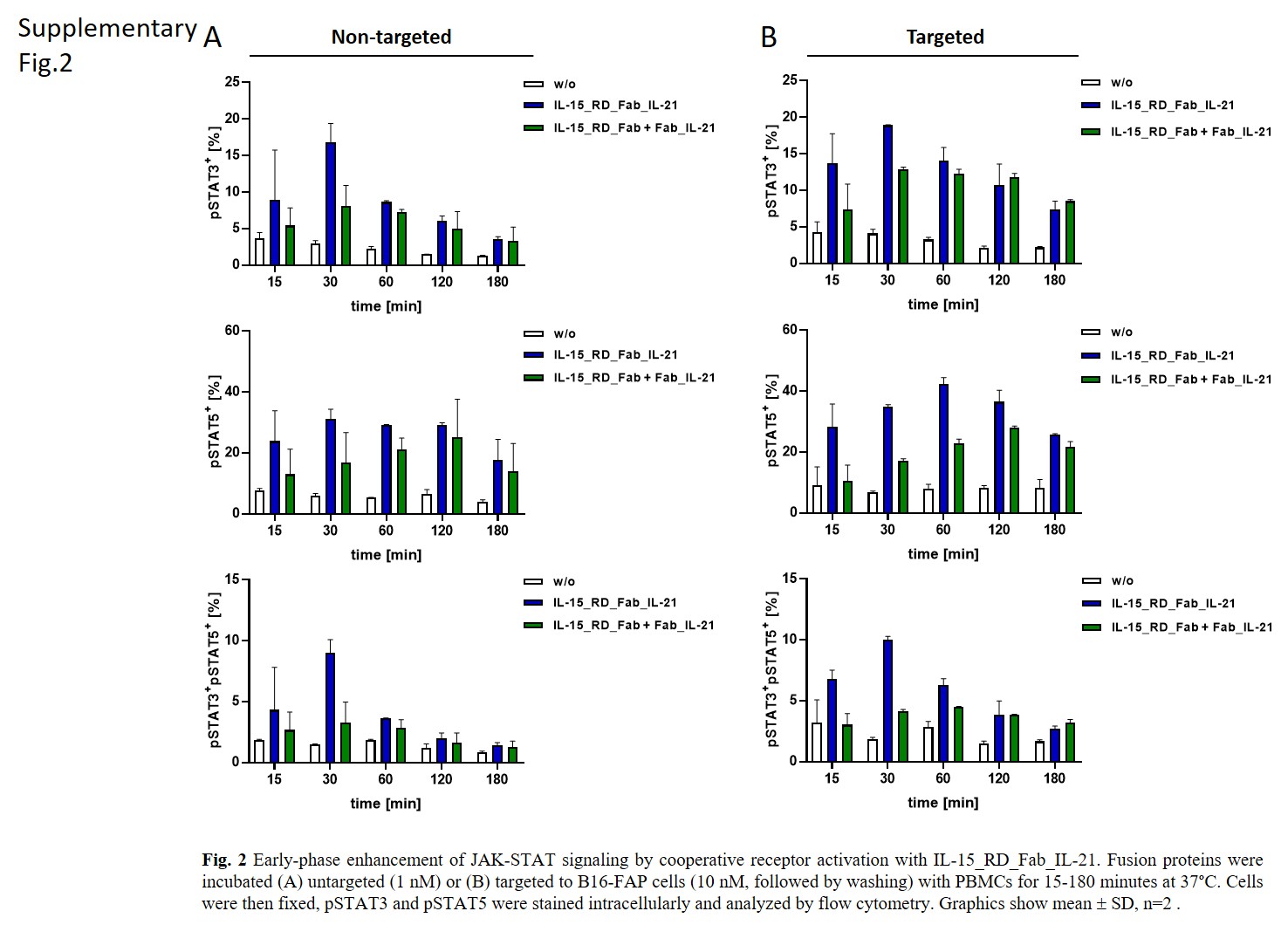

Supplement: Supplementary file 2 [file Image2.jpeg]
