# Supplementary material for: Trifunctional antibody-cytokine fusion protein formats for tumor-targeted combination of IL-15 with IL-7 or IL-21
Source: Front Immunol. 2025 Apr 30;16:1498697. doi: 10.3389/fimmu.2025.1498697 (PMC12075275; doi:10.3389/fimmu.2025.1498697)
Supplement: Supplementary file 3 [file Table1.pdf]

Supplementary Material

Table 1 Bi- and trifunctional antibody-cytokine fusion proteins – MW (calculated from sequence) and linker information

**Bifunctional fusion protein**	**MW (kDa)**	**Linker sequences**
scFv_RD_IL-15	51.4	L_1: SG_4SG_4 / L_2: $G_3SG_4SG_3SG_4SLQ$
scFv_IL-7	45.7	L_1: $A_3H_6G_4S$
scFv_IL-21	43.7	L_1: $A_3H_6G_4S$
Fab_IL-21	64.9	L_1: $G_2SG_4S_2G$
IL-15_RD_Fab	72.2	L_1: $G_3SG_4SG_3SG_4SLQ$ / L_2:GSG_3SG_4
**Trifunctional fusion protein**		
scFv_RD_IL-15_IL-7	69.0	L_1: SG_4SG_4 / L_2: $G_3SG_4SG_3SG_4SLQ$ / L_3: $A_3H_6G_4S$
RD_IL-15_scFv_IL-7	69.1	L_1: $G_3SG_4SG_3SG_4SLQ$ / L_2:$G_3SG_3SSG_3S$ / L_3: $A_3H_6G_4S$
scFv_RD_IL-15_IL-21	67.1	L_1: SG_4SG_4 / L_2: $G_3SG_4SG_3SG_4SLQ$ / L_3: $A_3H_6G_4S$
RD_IL-15_scFv_IL-21	67.1	L_1: $G_3SG_4SG_3SG_4SLQ$ / L_2:$G_3SG_3SSG_3S$ / L_3: $A_3H_6G_4S$
IL-15_RD_Fab_IL-21	88.3	L_1: $G_3SG_4SG_3SG_4SLQ$ / L_2:GSG_3SG_4 / L_3: $G_2SG_4S_2G$

scFv: single-chain fragment variable; Fab: fragment antigen binding; Linker are indicated in the name as underline character

Number of glycosylation sites:

IL-15 (UniProtKB P40933): 1 N-linked site; RD (IL-15Rα(Q13261) 31-107): 2 O-linked sites

IL-7 (UniProtKB P13232): 3 N-linked sites

IL-21 (UniProtKB Q9HBE4-1): 1 N-linked site
