# Supplementary material for: Trifunctional antibody-cytokine fusion protein formats for tumor-targeted combination of IL-15 with IL-7 or IL-21
Source: Front Immunol. 2025 Apr 30;16:1498697. doi: 10.3389/fimmu.2025.1498697 (PMC12075275; doi:10.3389/fimmu.2025.1498697)
Supplement: Supplementary file 4 [file Table2.pdf]

Supplementary Material

Table 2 Binding capacity of bi-and trifunctional antibody-cytokine fusion proteins to B16-FAP cells. EC_{50} values calculated from experimental data in Fig.1 C

**Fusion protein**	**EC_{50} ± SD (nM)**
scFv_RD_IL-15	15.04 ± 1.55
scFv_IL-7	1.95 ± 0.42
scFv_IL-21	15.80 ± 1.51
RD_IL-15_scFv_IL-7	5.56 ± 1.71
scFv_RD_IL-15_IL-21	4.86 ± 1.43
RD_IL-15_scFv_IL-21	7.67 ± 3.21
